# Targeted Re-Sequencing Approach of Candidate Genes Implicates Rare Potentially Functional Variants in Tourette Syndrome Etiology

**DOI:** 10.3389/fnins.2016.00428

**Published:** 2016-09-21

**Authors:** John Alexander, Hera Potamianou, Jinchuan Xing, Li Deng, Iordanis Karagiannidis, Fotis Tsetsos, Petros Drineas, Zsanett Tarnok, Renata Rizzo, Tomasz Wolanczyk, Luca Farkas, Peter Nagy, Urszula Szymanska, Christos Androutsos, Vaia Tsironi, Anastasia Koumoula, Csaba Barta, TSGeneSEE, Paul Sandor, Cathy L. Barr, Jay Tischfield, Peristera Paschou, Gary A. Heiman, Marianthi Georgitsi

**Affiliations:** ^1^Department of Molecular Biology and Genetics, Democritus University of Thrace Alexandroupoli, Greece; ^2^Department of Genetics, Rutgers, The State University of New Jersey Piscataway, NJ, USA; ^3^Human Genetics Institute of New Jersey, Rutgers, The State University of New Jersey Piscataway, NJ, USA; ^4^Computer Science Department, Purdue University West Lafayette, USA; ^5^Vadaskert Clinic for Child and Adolescent Psychiatry Budapest, Hungary; ^6^Department of Clinical and Experimental Medicine, University of Catania Catania, Italy; ^7^Department of Child Psychiatry, Medical University of Warsaw Warsaw, Poland; ^8^Child and Adolescent Psychiatry Clinic, Sismanoglio General Hospital of Attica Athens, Greece; ^9^Molecular Biology and Pathobiochemistry, Institute of Medical Chemistry, Semmelweis University Budapest, Hungary; ^10^ Tourette Syndrome Genetics — Southern and Eastern Europe Initiative (TSGeneSEE Consortium); ^11^Department of Psychiatry, University of Toronto Toronto, ON, Canada; ^12^Genetics and Development Division, Krembil Research Institute, University Health Network Toronto, ON, Canada; ^13^Program in Neurosciences and Mental Health, The Hospital for Sick Children Toronto, ON, Canada; ^14^Laboratory of General Biology, Department of Medicine, Aristotle University of Thessaloniki Thessaloniki, Greece

**Keywords:** next generation sequencing, targeted re-sequencing, genetic susceptibility, rare variants, TS candidate genes, *HDC*, *SLITRK1*

## Abstract

Although the genetic basis of Tourette Syndrome (TS) remains unclear, several candidate genes have been implicated. Using a set of 382 TS individuals of European ancestry we investigated four candidate genes for TS (*HDC, SLITRK1, BTBD9*, and *SLC6A4*) in an effort to identify possibly causal variants using a targeted re-sequencing approach by next generation sequencing technology. Identification of possible disease causing variants under different modes of inheritance was performed using the algorithms implemented in VAAST. We prioritized variants using Variant ranker and validated five rare variants via Sanger sequencing in *HDC* and *SLITRK1*, all of which are predicted to be deleterious. Intriguingly, one of the identified variants is in linkage disequilibrium with a variant that is included among the top hits of a genome-wide association study for response to citalopram treatment, an antidepressant drug with off-label use also in obsessive compulsive disorder. Our findings provide additional evidence for the implication of these two genes in TS susceptibility and the possible role of these proteins in the pathobiology of TS should be revisited.

## Introduction

Tourette Syndrome (TS; OMIM #137580) is a complex neurodevelopmental disorder of childhood onset, characterized by motor and vocal tics. It often presents with co-morbidities such as attention deficit hyperactivity disorder (ADHD) and/or obsessive-compulsive disorder (OCD), as well as mood disorders, anxiety and sleep disorders. Apart from various environmental factors that contribute to TS complexity (Hoekstra et al., 2013; Mathews et al., 2014), twin and family studies have long established that TS bears a strong genetic component (Price et al., 1985; Pauls et al., 1991, 2014). However, despite extensive on-going genetic research, concrete evidence supporting specific pathogenetic mechanisms is still largely lacking. Several genes (such as *SLITRK1, HDC, NLGN4, CNTNAP2, IMMP2L, SLC6A4* also known as *SERT*, and *NTN4*) and chromosomal loci have been implicated to date although, associations between genetic variation and TS are often limited to specific population-based cohorts or may be restricted to extremely rare mutations identified solely in unique multigenerational pedigrees.

A very promising candidate TS susceptibility region was implicated for the first time in 2005 by Abelson et al. (Abelson et al., 2005). The study reported a *de novo* inversion in a TS patient, occurring in the vicinity of the Slit and Trk-like family member 1 (*SLITRK1*) gene. The authors subsequently sequenced *SLITRK1* in 174 unrelated TS probands and identified rare mutations. This spurred intense interest in TS genetics with multiple studies reporting rather mixed results (Abelson et al., 2005; Deng et al., 2006; Keen-Kim et al., 2006; Wendland et al., 2006; Chou et al., 2007; Scharf et al., 2008; Zimprich et al., 2008; O'Roak et al., 2010; Karagiannidis et al., 2012). Recently, our group and others have provided evidence for association of the disorder with common alleles and haplotypes being over-transmitted in TS cases; these findings support the hypothesis of the existence of an as of yet unidentified risk factor in linkage disequilibrium (LD) with the associated markers and possibly lying in gene regulatory regions (Miranda et al., 2009; Karagiannidis et al., 2012).

Abnormalities of cortico-striatal-thalamic-cortical pathways and dysfunction of both dopamine and serotonin neurotransmitter systems have long been considered in association to TS (Peterson et al., 2003; Kalanithi et al., 2005; Muller-Vahl et al., 2005). The recent implication of the *L-histidine decarboxylase* (*HDC*) gene in TS etiology has also raised the intriguing hypothesis of the involvement of histaminergic neural pathways in the onset of the disorder (Ercan-Sencicek et al., 2010; Lei et al., 2012). Moreover, a genome-wide scan for *de novo* or transmitted rare copy number variants in TS had found enrichment of genes within the histamine signaling pathways (Fernandez et al., 2012). Subsequently, our group found evidence for over-transmission of *HDC* alleles and significantly associated haplotypes in trios of European origin (Karagiannidis et al., 2013).

The serotonin transporter gene (*SERT*, 5-HTT), coding for a solute carrier family 6 member 4 protein (*SLC6A4*) was reported to carry both common and rare variants as well as a high-expressing haplotype associated with increased gene expression (5-HTTLPR/rs25531/rs25532) and protein activity (p.I425V), thus potentially contributing to serotonergic abnormalities in TS with or even more so, without OCD (Kilic et al., 2003; Moya et al., 2013). These high-expressing alleles have been previously significantly associated with OCD (Hu et al., 2006; Wendland et al., 2008), which is believed to share a degree of common genetic background with TS (Mathews and Grados, 2011; Yu et al., 2015).

Variants in the BTB/POZ domain-containing protein 9 gene (*BTBD9*) have been previously associated with restless legs syndrome (RLS) (Allen et al., 2005; Winkelmann et al., 2007). High RLS prevalence has also been reported in TS and, similar to TS, it has been linked to frontostriatal circuits dysfunction and is responsive to dopamine neurotransmission modification (Turjanski et al., 1999). *BTBD9* variants were found to be associated with TS in French Canadian and Chinese patient cohorts (Riviere et al., 2009; Guo et al., 2012). Yet, all analysed variants were intronic and thus do not provide direct functional relevance; however, they could be in LD with potentially functional yet unknown variants, such as variants in gene regulatory regions or, tissue and neuroanatomical region-specific, naturally-occurring somatic mutations (Lodato et al., 2015).

The abovementioned TS susceptibility genes have emerged from candidate gene association studies (*SLC6A4, BTBD9*), chromosomal aberration studies (*SLITRK1*), and linkage analysis studies (*HDC*). Despite decades of efforts in the field of TS genetics, the overall number of identified variants likely predisposing to TS remains extremely small. With the advent of next-generation sequencing technology, there is a resurging interest toward the identification of rare variants of potentially intermediate-to-high penetrance effects that might aid in explaining part of the missing heritability of the TS phenotype.

In the present study we report results on a targeted re-sequencing approach in a cohort of 382 TS cases of European and Canadian origin, aiming to explore the existence of rare genetic variation across four genes that have attracted considerable interest in the past years, namely *SLITRK1, HDC, BTBD9*, and *SLC6A4*. Moreover, given the findings from our previous studies on association of *SLITRK1* and *HDC* haplotypes with TS (Karagiannidis et al., 2012, 2013), we wanted to explore whether previously associated risk haplotypes are in linkage with rare coding, and thus directly functional variants in these two genes.

## Materials and methods

### Study subjects

A total of 382 individuals with TS were included in the study. The subjects represent affected cases from family trios who had been previously recruited within The Tourette Syndrome Genetics—Southern and Eastern Europe Initiative (TSGeneSEE) and originated from Hungary (*n* = 117), Italy (*n* = 95), Poland (*n* = 68), Greece (*n* = 17), and Albania (*n* = 8). In addition, 77 TS cases originating from Canada were also available. The majority of these individuals have been described previously (Karagiannidis et al., 2013). Assessment was performed by on-site clinicians using the tools provided by the TS Association International Consortium for Genetics (TSAICG, 2007). TS was ascertained according to DSM-IV-TR criteria for Italy, Hungary, Albania, and Greece, and DSM-IV for Poland. For Canadian samples, TS was ascertained using DSM-III-R criteria. Differences between DSM-III-R, DSM-IV, and DSM-IV-TR are minimal; the upper age limit of onset is 18 in DSM-IV (and DSM-IV-TR) and 21 in DSM-III-R, and the “marked distress” criterion, possibly pointing to more severe cases, only appears in DSM-IV (applied only to the Polish cases; Cath et al., 2011). For all samples, collection was approved by local Ethics Boards and written informed consent was taken from all participating individuals or their parents.

### Genomic DNA extraction

Peripheral blood samples were collected in EDTA-containing tubes and genomic DNA was purified using the Qiagen Gentra Puregene kit with minor protocol modifications (Qiagen GmbH, Hilden, Germany).

### Exome capture and targeted re-sequencing

Using the Fluidigm custom panel design pipeline, the four candidate genes (*HDC, SLITRK1, SLC6A4, BTBD9*) were targeted for sequencing and custom primers were designed. For *HDC* and *SLITRK1*, the whole locus including 1 kb upstream and downstream were targeted. For *SLC6A4* and *BTBD9*, exonic regions of the genes were targeted (Table S1).

DNA samples were tagged and amplified using Fluidigm Access Array System (Fluidigm Corporation, South San Francisco, USA). The 48.48 Access Array integrated fluidics circuit (IFC) protocol was followed. The sample mix solution was prepared using 50 ng/uL of genomic DNA. The gDNA was then combined with sample pre-mix and loaded into the 48.48 Access Array IFC. Upon amplification completion, the IFC was placed on the Post-PCR IFC Controller AX and the Harvest (151x) Script was run. After the completion of the script 10 uL of the product was removed and placed into a 96-well PCR plate. Barcoding PCR was then set up using the manufacturer's instructions on a conventional PCR thermal cycler. With the incorporation of a unique identifier or barcode for each sample and the necessary sequencing adaptors, it is possible to process all samples simultaneously on the sequencing platform.

Quality and quantity of the library were evaluated on the Agilent BioAnalyzer (Agilent Technologies, Santa Clara, CA, US) and quantified using KAPA quantification. The sequencing was then completed following the protocol for the Illumina (Illumina, San Diego, CA) MiSeq V3 chemistry 2 × 100-bp paired-end reads. Once the sequencing was completed, de-multiplexing was performed using Illumina's bcl2fastq2 v2.15 to generate sample specific fastq files.

### Sequence alignment and genotype calling

Sequence alignment was performed using Burroughs Wheeler alignment (BWA; Li and Durbin, 2009) using the reference human genome build hg19 from UCSC. Samtools (Li et al., 2009) was used to convert BAM alignment files to SAM format and then sort and index them. INDEL realignment was performed using IndelRealigner and base quality score recalibration was performed using BaseRecalibrator from GATK (McKenna et al., 2010). PCR and optical duplicates were removed from samples using Picard (http://picard.sourceforge.net). Variant calling was done on the targeted regions (Table S1) by combining all the samples using the GATK pipeline and following their best practices protocol. Further filtering was applied to keep only the variants that passed the quality control filters and 95% genotype call rate. Sequencing data (BAM files) can be accessed via http://paschou-lab.mbg.duth.gr/share_data.html.

### Variant ranker analysis

Variants were then annotated using ANNOVAR (Wang et al., 2010) and ranked using Variant Ranker tool (http://paschou-lab.mbg.duth.gr/Software.html). Variant ranker aggregates annotation information based on allelic frequency scores, conservation scores, prediction algorithm scores, and clinical information. Using Variant Ranker, the variants are prioritized on the basis of their effect, novelty, and annotation information. A variant was designated as novel if not present in database of human variation dbSNP build 138 and had a minor allele frequency (MAF) ≤ 0.01 in 1000 Genomes, Exome Aggregation Consortium (ExAC) and National Heart, Lung, and Blood Institute Exome Sequencing Project (NHLBI ESP6500) database.

### VAAST analysis

Scoring and prioritization analysis of deleterious alleles was performed using VAAST 2.0 (Hu et al., 2013). We applied the likelihood ratio test in VAAST using both dominant and recessive disease models of inheritance, with the help of a background file comprising of 189 genomes from 1000 Genomes project (Abecasis et al., 2012), 184 Danish exomes (Li et al., 2010), 10Gen genome dataset (Reese et al., 2010), and 40 whole genomes from the Complete Genomics Diversity Panel.

### Identification of linkage disequilibrium (LD) SNPs

We used the web-based application LDproxy (Machiela and Chanock, 2015) to identify proxy LD SNPs and LdOOKUP (http://purces04.u.hpc.mssm.edu/ldookup/ldookup.cgi) to browse for variants in LD with other GWAS studies from the Psychiatric Genomics Consortium, brain eQTL data and the NHGRI GWAS catalog (Welter et al., 2014).

### Sanger sequencing

PCR primers were designed using PRIMER3 (http://primer3plus.com) and confirmed to have unique genomic product by UCSC *in-silico* PCR (http://genome.ucsc.edu/cgi-bin/hgPcr), targeting amplicons of sizes between 300 and 400 bp. PCR products were purified using QIAquick PCR purification kit from Qiagen (QIAGEN, CA), followed by Sanger sequencing on an ABI 3730xl sequencer in Genscript (NJ, USA), using the BigDye Terminator v.3.1 chemistry.

## Results

### Identification of variants

We explored whether coding variations in four genes, namely, *HDC, SLITRK1, BTBD9* and *SLC6A4* play a role in TS susceptibility. We utilized next-generation sequencing technology to perform targeted sequencing on a total of 60 kilobases, including the entire *HDC* and *SLITRK1* genes, and spanning 15 exons of *SLC6A4* and 11 exons of *BTBD9* genes, across 382 TS cases of Caucasian origin (Table S1). Regarding data quality, more than 95% of bases were sequenced with >99.9% accuracy, covering most amplicons at >100X.

We obtained 336 single nucleotide polymorphisms (SNPs) with 31 exonic SNPs (see summary statistics on Table 1). We focused only on functionally important non-synonymous exonic variants and pursued validation via Sanger sequencing. We found a novel rare (MAF = 0.13%) variant in *HDC* residing in exon 12 (*HDC*:NM_002112:exon12:c.A1564C:p.I522L), two rare (MAF = 0.13%) novel *SLITRK1* variants, at positions chr13:g.84454582 (*SLITRK1*:NM_052910 :exon1:c.G1061C:p.G354A). and chr13:g.84454485 (*SLITRK1*:NM_052910:exon1:c.G115 8T:p.K386N), residing in exon 1 of the gene, and two known rare variants, rs146746846 (MAF = 0.13%) (*SLITRK1*:NM_052910:exon1:c.C892T:p.H298Y) and rs150504822 (MAF = 0.26%; *SLITRK1*:NM_052910:exon1:c.A1252T:p.T418S), also in exon 1 (Table 2). The five confirmed variants were ranked within the top six potentially etiologically relevant variants by our Variant Ranker algorithm and were all predicted to be deleterious by MutationTaster functional prediction algorithm (Schwarz et al., 2010; Table S2). One confirmed *SLITRK1* variant (rs146746846) was also identified by VAAST under a recessive model of inheritance (Table S3). Proxy LD SNPs available for rs150504822 on *SLITRK1* were input into LdOOKUP to identify significant variants in other GWAS studies. A variant in LD with rs150504822, namely rs6563353, was identified (Tables S4, S5); rs6563353 has been positively associated (*p* = 2.06e-6) with increased citalopram-induced general side effect burden, in patients with depression (Adkins et al., 2012). Citalopram is an antidepressant drug of the selective serotonin reuptake inhibitor class, with an off-label use for OCD treatment.

**Table 1:** **Summary of functional annotation of identified variants**.

**Variant category**	**Number of variants**
Upstream	23
5′UTR	9
Coding (Exonic)	31
Intronic	205
3′UTR	40
Downstream	17
Intergenic	11
Total	336
Nonsynonymous	17
Synonymous	14
Total Coding	31

*UTR, Untranslated region*.

**Table 2:** **Nonsynonymous variants confirmed by Sanger sequencing**.

**Location**	**Ref/Alt**	**Het**	**Gene**	**RefGene variant annotation**	**1000G**	**LRT**	**MutationTaster**
chr13:84454582	C/G	1	*SLITRK1*	*SLITRK1*:NM_052910:exon1:c.G1061C:p.G354A	NA	D	D
chr13:84454485	C/A	1	*SLITRK1*	*SLITRK1*:NM_052910:exon1:c.G1158T:p.K386N	NA	D	D
chr13:84454751(rs146746846)	G/A	1	*SLITRK1*	*SLITRK1*:NM_052910:exon1:c.C892T:p.H298Y	NA	D	D
chr13:84454391(rs150504822)	T/A	2	*SLITRK1*	*SLITRK1*:NM_052910:exon1:c.A1252T:p.T418S	0.00019	D	D
chr15:50534882	T/G	1	*HDC*	*HDC*:NM_002112:exon12: c.A1564C:p.I522L	NA	N	D

*Chr, chromosomal location; Ref, Reference allele; Alt, Alternative (rare) allele; Het, Number of heterozygous cases with the variant; 1000G, 1000 Genomes Project variant frequency (if available); LRT, Likelihood Ratio Test; LRT D, Deleterious; N, Neutral; MutationTaster D, Disease-causing; NA, Not available*.

## Discussion

To our knowledge, this is the first study applying next generation sequencing technology in quest for genetic susceptibility variants shaping the genomic landscape of TS. It is expected that at least part of the missing heritability of complex disorders, such as TS, will be attributable to low-frequency variants with intermediate penetrance effects (Manolio et al., 2009) and, if truly causative, they could account for a small proportion of TS cases.

In the present study, we identified and confirmed a rare novel *HDC* coding variant as well as two rare novel and two rare known *SLITRK1* coding variants, all confirmed by Sanger sequencing (Table 2). We did not extensively cover the regulatory regions of the genes; thus, regulatory variants may have been missed resulting in under-estimation of the contribution of these genes to TS risk.

The recent implication of the *L-histidine decarboxylase* (*HDC*) gene in TS etiology has raised the intriguing hypothesis of the involvement of histaminergic neural pathways in the onset of the disorder. Ercan-Sencicek et al. studied a family with a father and all eight of his children affected with TS, and found a rare premature termination codon mutation (p.W317X) in *HDC*, also absent in 3,000 control individuals. Due to the demonstrated dominant-negative mode of function of the mutant HDC enzyme, the authors concluded that histaminergic neurotransmission is most likely diminished in their patients (Ercan-Sencicek et al., 2010). However, *HDC* coding variants seem to be extremely rare since apart from the mutation identified in the original pedigree, *HDC* has not been found altered in other TS cases of Caucasian or Asian origin (Ercan-Sencicek et al., 2010; Lei et al., 2012). Interestingly, a preceding genome-wide study of 95 French Canadian trios with familial history of TS (Riviere et al., 2010) had also shown association to a microsatellite marker on chromosome 15 (D15S1016), lying within the same interval found to be linked with TS in the original study that implicated *HDC* in TS etiology. Finally, a recent genome-wide scan for *de novo* or transmitted rare copy number variants in TS found enrichment of genes within the histamine receptor signaling pathways (Fernandez et al., 2012). Even though in the present study we did not detect the rare variants previously reported in the literature, we did find a novel exonic nonsynonymous variant, chr15:g.50534882 (c.A1564C:p.I522L), in one TS case.

Histamine plays a central role in gastric acid secretion, innate, and acquired immunity and immunomodulation, bronchoconstriction, vasodilation and neurotransmission. The neuronal histaminergic system is involved in a number of basic physiological functions such as circadian rhythmicity, energy metabolism, neuro-endocrine homeostasis, stress, sensory, and motor functions, cognition, attention, learning, and memory. Histidine decarboxylase (HDC) is the key enzyme for histamine production from histidine and in the brain its mRNA is expressed exclusively in the posterior hypothalamus (Haas et al., 2008). So far, the histaminergic system has not received as much attention as other monoaminergic systems of the brain. Classically established as a “peripherally” important mediator of inflammation, the importance of histamine in neurotransmission, and its role in neuropsychiatric disorders is only recently starting to become appreciated (Tiligada et al., 2011) and deserves further investigation also in the context of TS. Interestingly, *Hdc*^−∕−^ deficient mice have several traits relevant to features of TS and have shown decreased brain histamine and increased sensitivity to stereotypic behaviors (i.e., hyperlocomotion) upon administration of dopamine agonists (Kubota et al., 2002) and repetitive grooming after induced fear (Xu et al., 2015). Such stimulant-induced movements, including rearing, sniffing, and biting have previously been proposed as a model of human tics (Saka and Graybiel, 2003; Castellan Baldan et al., 2014).

*SLITRK1* has been extensively studied since its identification as a much promising TS candidate gene. However, with the exception of the variants reported by Abelson et al. (2005), additional novel non-synonymous mutations have not been identified in a large number of subsequent studies interrogating patient cohorts (Deng et al., 2006; Chou et al., 2007; Scharf et al., 2008; Zimprich et al., 2008) or single families (Robertson and Orth, 2006; Verkerk et al., 2006; Fabbrini et al., 2007; Pasquini et al., 2008). On the other hand, genetic association studies and haplotype analyses could not rule out the implication of *SLITRK1* in TS etiology either via the existence of risk factors in LD with *SLITRK1* (Miranda et al., 2009) or as of yet unidentified SLITRK1 regulatory variants (Karagiannidis et al., 2012). In our study, we did not detect the SLITRK1 variants that had been previously reported in association to TS but we report on the identification of two novel variants, chr13:g.84454582 and chr13:g.84454485, each found in one TS patient. We also identified two rare non-synonymous SLITRK1 variants (rs150504822 and rs146746846, identified in two TS cases and one TS case, respectively). Interestingly, rs150504822 was found in LD with rs6563353, a variant that has been previously associated with citalopram-induced general side-effect burden in patients with depression (Adkins et al., 2012). Citalopram, is a well-tolerated acute treatment drug conferring positive response in children and adolescents with OCD (Thomsen et al., 2001; Mukaddes et al., 2003).

Interestingly, *SLITRK1* encodes a transmembrane protein necessary for dendritic growth, axon guidance and branching of neuronal cells. It is expressed in both the embryonic and postnatal brain (cortex, thalamus, and basal ganglia), reflecting neuroanatomical regions most commonly affected in TS (Proenca et al., 2011), as well as in mature neurons with large axons (Aruga and Mikoshiba, 2003). *Slitrk1*^−∕−^ mice exhibited elevated anxiety and depression-like behaviour, consistent with TS symptoms, in which the anxiety-like behaviour was attenuated upon clonidine administration, a drug often used in TS treatment (Katayama et al., 2010).

Our previous and present studies suggest that *SLITRK1* and *HDC* may have an under-appreciated role in TS aetiology. Unarguably, the functional role of the identified variants warrants further investigation by a wide array of experiments. Next-generation sequencing technology is rapidly becoming a routine, albeit still costly approach to target patient genomes that is expected to significantly expedite the quest for rare variants with reduced penetrance, not only in TS susceptibility but also other complex neurodevelopmental and neuropsychiatric disorders.

## Author contributions

JA, HP, JX, LD, and IK performed experimental procedures, data analysis and interpretation, and participated in manuscript writing. FT and PD participated in data analysis and interpretation, and in manuscript writing. ZT, RR, TW, LF, PN, US, CA, VT, AK, CB, PS, and CLB performed patient sample recruitment and ascertainment, and participated in data analysis and interpretation. JT, PP, GH, and MG designed the study, supervised experimental procedures, performed data analysis and interpretation, and wrote the manuscript. All authors read and approved the final version to be published and agreed to be accountable for all aspects of the work.

### Conflict of interest statement

The authors declare that the research was conducted in the absence of any commercial or financial relationships that could be construed as a potential conflict of interest.
